# Weighted gene co-expression network analysis identifies specific modules and hub genes related to coronary artery disease

**DOI:** 10.1038/s41598-021-86207-0

**Published:** 2021-03-23

**Authors:** Peng-Fei Zheng, Lu-Zhu Chen, Yao-Zong Guan, Peng Liu

**Affiliations:** 1 Department of Cardiology, The Central Hospital of Shao Yang, 36 QianYuan lane, Shaoyang, 422000 Hunan People’s Republic of China; 2 Graduate School of Guangxi Medical University, 22 Shuangyong Road, Nanning, 530021 Guangxi People’s Republic of China

**Keywords:** Atherosclerosis, Coronary artery disease and stable angina

## Abstract

This investigation seeks to dissect coronary artery disease molecular target candidates along with its underlying molecular mechanisms. Data on patients with CAD across three separate array data sets, GSE66360, GSE19339 and GSE97320 were extracted. The gene expression profiles were obtained by normalizing and removing the differences between the three data sets, and important modules linked to coronary heart disease were identified using weighted gene co-expression network analysis (WGCNA). Gene Ontology (GO) functional and Kyoto Encyclopedia of Genes and genomes (KEGG) pathway enrichment analyses were applied in order to identify statistically significant genetic modules with the Database for Annotation, Visualization and Integrated Discovery (DAVID) online tool (version 6.8; http://david.abcc.ncifcrf.gov). The online STRING tool was used to construct a protein–protein interaction (PPI) network, followed by the use of Molecular Complex Detection (MCODE) plug-ins in Cytoscape software to identify hub genes. Two significant modules (green-yellow and magenta) were identified in the CAD samples. Genes in the magenta module were noted to be involved in inflammatory and immune-related pathways, based on GO and KEGG enrichment analyses. After the MCODE analysis, two different MCODE complexes were identified in the magenta module, and four hub genes (*ITGAM*, degree = 39; *CAMP*, degree = 37; *TYROBP*, degree = 28; *ICAM1*, degree = 18) were uncovered to be critical players in mediating CAD. Independent verification data as well as our RT-qPCR results were highly consistent with the above finding. *ITGAM*, *CAMP*, *TYROBP* and *ICAM1* are potential targets in CAD. The underlying mechanism may be related to the transendothelial migration of leukocytes and the immune response.

## Introduction

Inflammation plays a crucial role in the pathophysiology of coronary artery disease (CAD), it is involved in the formation, erosion and final rupture of atherosclerotic plaque, resulting in partial or total occlusion of coronary artery. This might result in myocardial ischemia and hypoxia and thereby an acute myocardial infarction (AMI)^1^. Complete occlusion usually leads to ST-elevation in the electrocardiogram, which is defined as an acute ST-segment elevation myocardial infarction (STEMI). Partial occlusion or occlusion with collateral circulation without ST-segment elevation is classified as unstable coronary syndrome. Unstable coronary syndromes without elevated Troponin T (TnT, a marker of myocardial necrosis) were defined as non-ST-segment elevation acute coronary syndromes, while those with elevated TnT were defined as non-ST-segment elevation myocardial infarction (non-STEMI)^2^. Although with the spread and popularization of emergency percutaneous coronary intervention (PCI) treatment, the prognosis of patients with AMI can be significantly improved by rapidly restoring blood flow in occluded vessels. However, CAD still maintains a high morbidity and mortality, and leading to significant reduction in quality of life of those patients as well as poses a hefty burden on healthcare systems^3,4^. The overall prevalence of CAD, also known as ischemic heart disease, nearly has risen steadily since 1990, reaching 182 million cases and 9.14 million deaths in 2019^5^. Well established risk factors for CAD include high blood pressure, diabetes, a sedentary lifestyle, smoking, family history, obesity, stress and hyperlipidemia. Although lots of efforts have been undertaken in recent years, the prevention and cure of CAD remains a daunting challenge for physicians around the world. There is an urgent need for further exploration of the potential molecular mechanisms correlated with CAD. Existing literature indicates that CAD is primarily mediated by coronary atherosclerosis^6^. Early intervention in preventing atherosclerosis could significantly decrease CAD, stroke and other ischemic diseases from occurring and developing^7^.

Microarray analysis might serve as a novel and practical approach to identify susceptibility genes correlated with coronary heart disease^8^. However, the reproducibility and sensitivity of microarray analysis based on differentially expressed genes may be limited^9,10^. Exactly, hence, currently, the microarray-based transcriptome analysis has been largely replaced by (or even singe cell-based) RNA seq. Furthermore, Gene co-expression network-based methods have been widely used in processing microarray^11,12^ and RNA seq data^13^ and have especially been used to identify meaningful functional modules. Weighted gene co-expression network analysis (WGCNA) is one of the most effective methods of gene co-expression network analysis. Transcriptome data from different sources within the same species can be grouped together for WGCNA analysis^14^. WCGNA generates a scale-free network of gene–gene interactions, if some genes always have similar expression changes in a physiological process or different tissues, and these genes will be enriched in a common significant module. Furthermore, it can be used to further analyze the correlation between modules and phenotypes or clinical characteristics^15^. Given the capabilities of WGCNA in formulating a co-expression network comprising of significant modules, we are able to glean new information regarding CAD features and may uncover novel insights in CAD-related molecular mechanisms, signaling pathways and genetic biomarkers.

## Results

### Data preprocessing

Interpatch difference removal and data normalization were carried out to obtain the final gene expression profiles. 113 samples yielded a total of 23,493 gene symbols. Further information regarding gene expression profile and the sample phenotypes are depicted in Supplementary Tables S1A,B and S2.

### Weighted gene co‑expression networks

**Figure 1:**
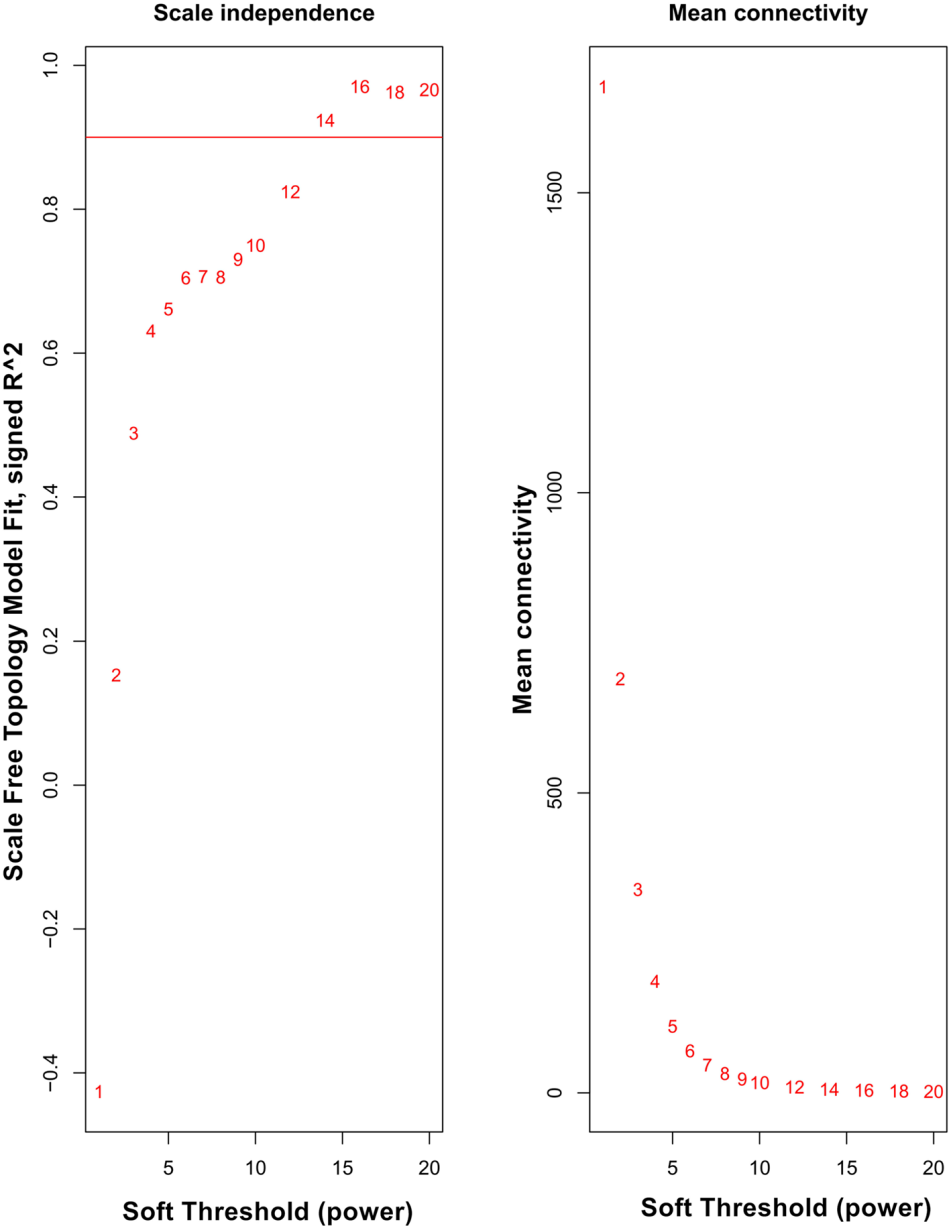
Analysis of network topology for various soft-thresholding powers. The left panel shows the scale-free fit index (y-axis) as a function of the soft-thresholding power (x-axis). The right panel displays the mean connectivity (degree, y-axis) as a function of the soft-thresholding power (x-axis).

**Figure 2:**
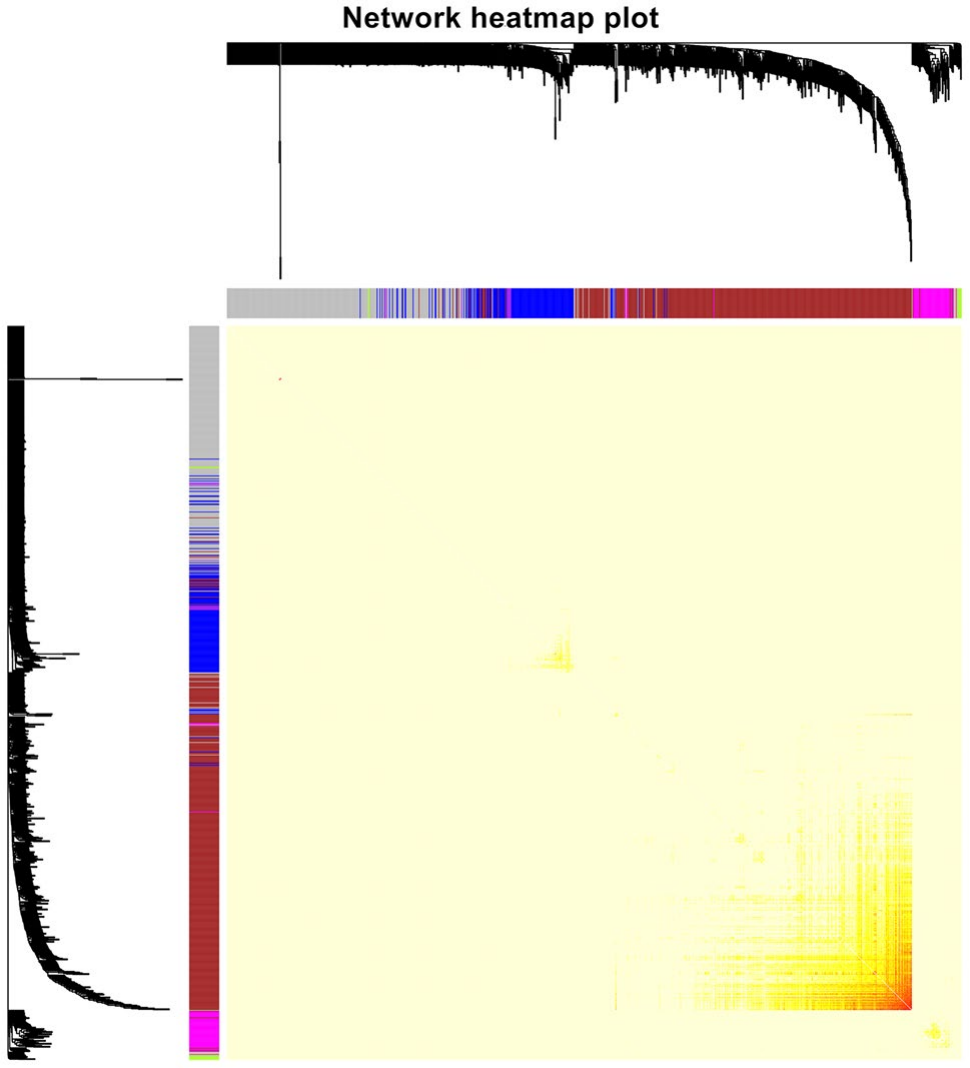
Heatmap plot of topological overlap in the gene network. In the heatmap, each row and column correspond to a gene, light color denotes low topological overlap, and progressively darker red denotes higher topological overlap. Darker squares along the diagonal correspond to modules. The gene dendrogram and module assignment are shown along the left and top.

**Figure 3:**
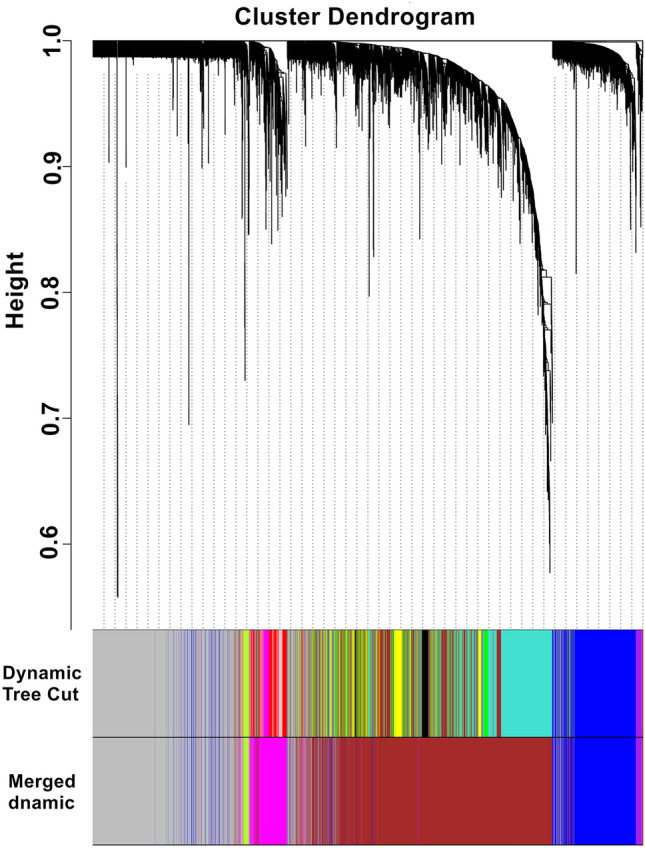
Clustering dendrogram of genes. Gene clustering tree (dendrogram) obtained by hierarchical clustering of adjacency-based dissimilarity. The colored row below the dendrogram indicates module membership identified by the dynamic tree cut method, together with assigned merged module colors and the original module colors.

Weighted gene co-expression networks were constructed based on identified genes after determining the soft threshold (β = 14) (Fig. 1). In order to create a topological overlap matrix (TOM), the adjacency and correlation matrices of the gene expression profile were calculated. A final gene clustering tree based on the gene–gene non-ω similarity was produced (Fig. 2). Using the hierarchical average linkage clustering method in combination with the TOM, we proceeded to identify gene modules of each gene network. The dynamic tree cut algorithm highlighted five gene modules (Fig. 3). Genes that did not fit in any modules were discarded from further analyses (presented as gray modules).

### Identification of the modules of interest

**Figure 4:**
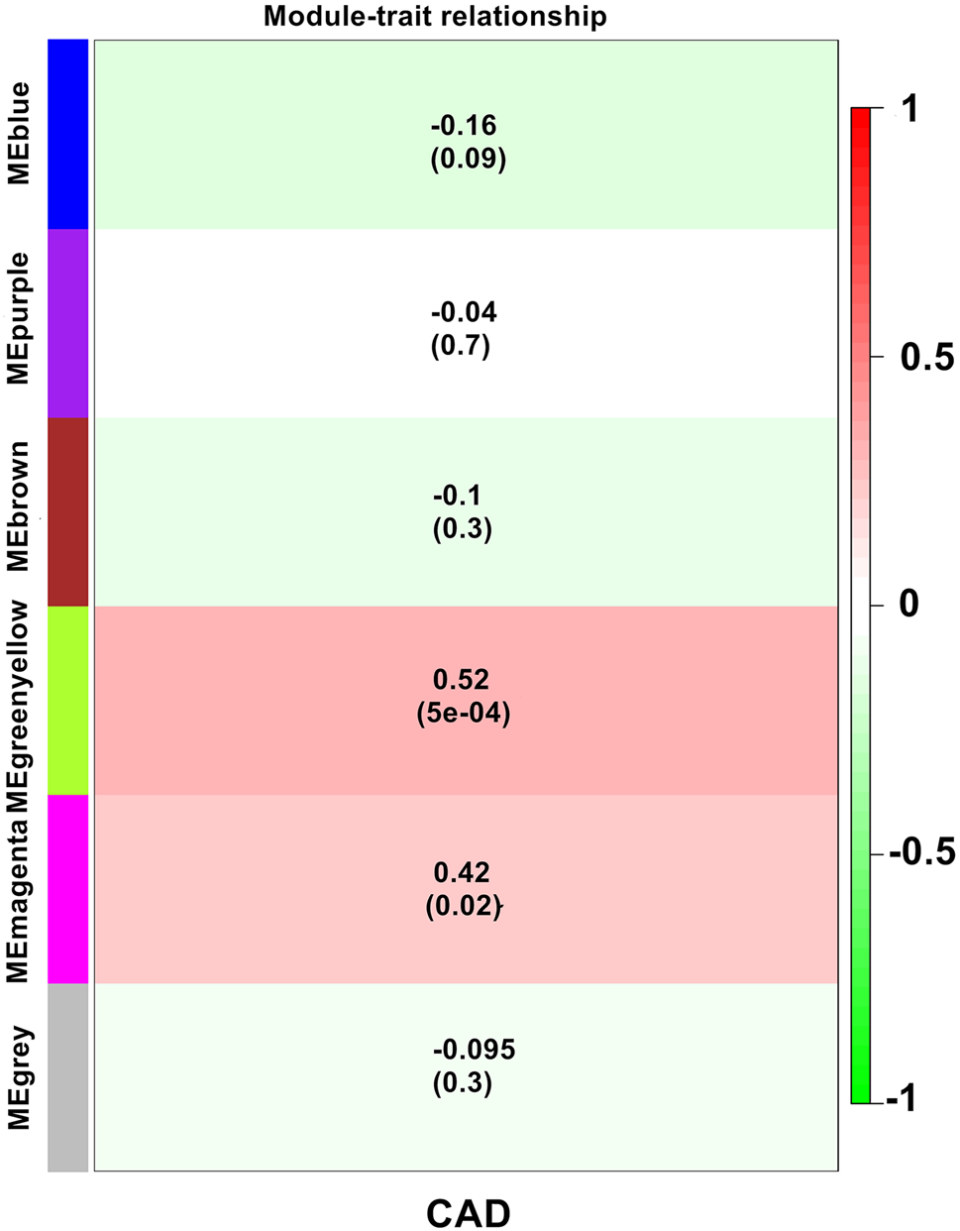
Module-feature associations. Each row corresponds to a modulEigengene and the column to the clinical phenotype. Each cell contains the corresponding correlation in the first line and the P-value in the second line. The table is color-coded by correlation according to the color legend.

Biologically significant modules which those that strongly correlated to clinicopathological features. Supplementary Fig. S1 and Fig. 4 show that the green–yellow (*r*^2^ = 0.52, *P* = 5E−04) and magenta (*r*^2^ = 0.42, *P* = 0.02) modules were highly correlated with CAD. Therefore, subsequent analyses were carried out on genes from both these modules. In addition, Supplementary Fig. S2 shows a highly significant correlation between gene significant (GS) versus module membership (MM) in the green-yellow (A) and magenta (B) modules with CAD.

### Module preservation test

We performed preservation analysis of the expression profiles of CAD and noticed that there were one weak and two strong preserved modules between CAD and control subjects (Supplementary Fig. S3). The statistical results of medianRank and Zsummary are consistent, which indicates that the module size has little effect on the preservation analysis. We noticed that the green-yellow and magenta modules were highly preserved with CAD; blue and brown modules were weakly preserved with CAD. These findings demonstrated that the gene expression patterns between the CAD and control subjects are different to a large extent.

### Enrichment analysis of interesting modules

**Figure 5:**
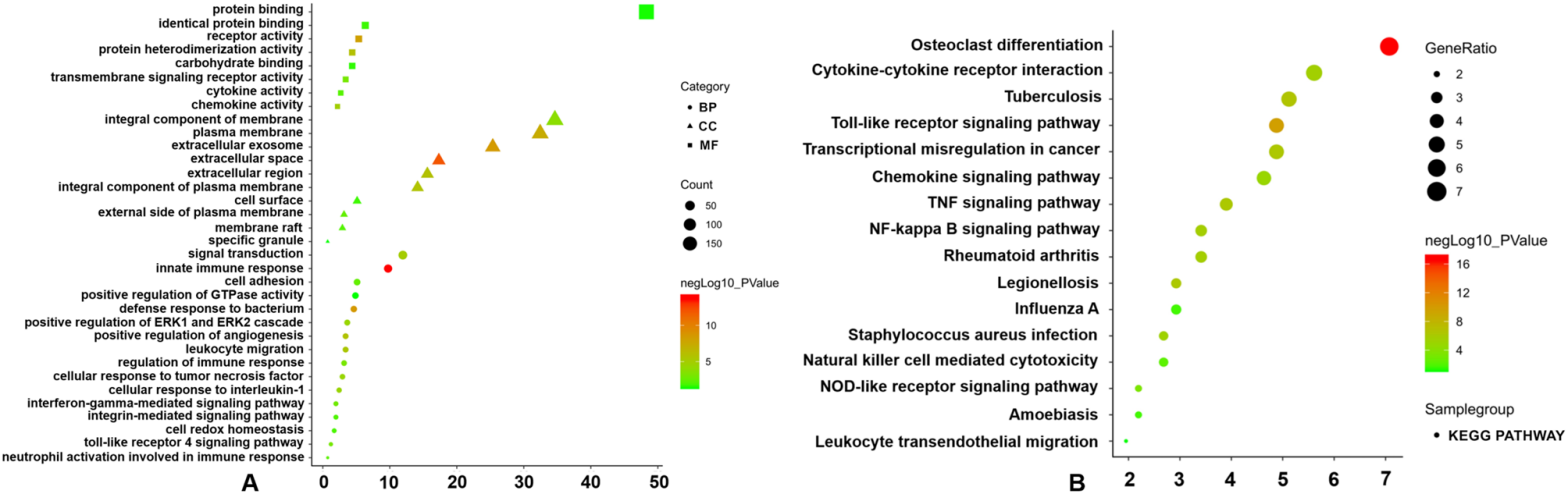
GO functional and KEGG pathway enrichment analyses for genes in the object module. The x-axis shows the number of genes and the y-axis shows the GO and KEGG pathway terms. The − log10 (P-value) of each term is colored according to the legend. (**A**) GO functional enrichment analysis. (**B**) KEGG pathway enrichment analysis^64^.

Biological functions of genes in both these modules were then subjected to further GO and KEGG pathway enrichment analyses. A total of 309 genes in the magenta module (Supplementary Table S3) were significantly correlated with the following pathways: leukocyte transendothelial migration signaling pathway (*ITGAM* and *ICAM1*), TNF signaling pathway (*ICAM1*), and the Staphylococcus aureus infection (*ITGAM* and *ICAM1*), rheumatoid arthritis (*ICAM1* and *ITGAM*), tuberculosis (*ITGAM* and *CAMP*), natural killer cell-mediated cytotoxicity (*ICAM1* and *TYROBP*), and NF-kappa B signaling pathways (*ICAM1*). The KEGG pathway analysis, molecular functions, biological processes as well as cellular components are depicted Fig. 5, with a more detailed presentation of data included in Supplementary Tables S4 and S5.

### PPI network construction and module analysis of DEGs

**Figure 6:**
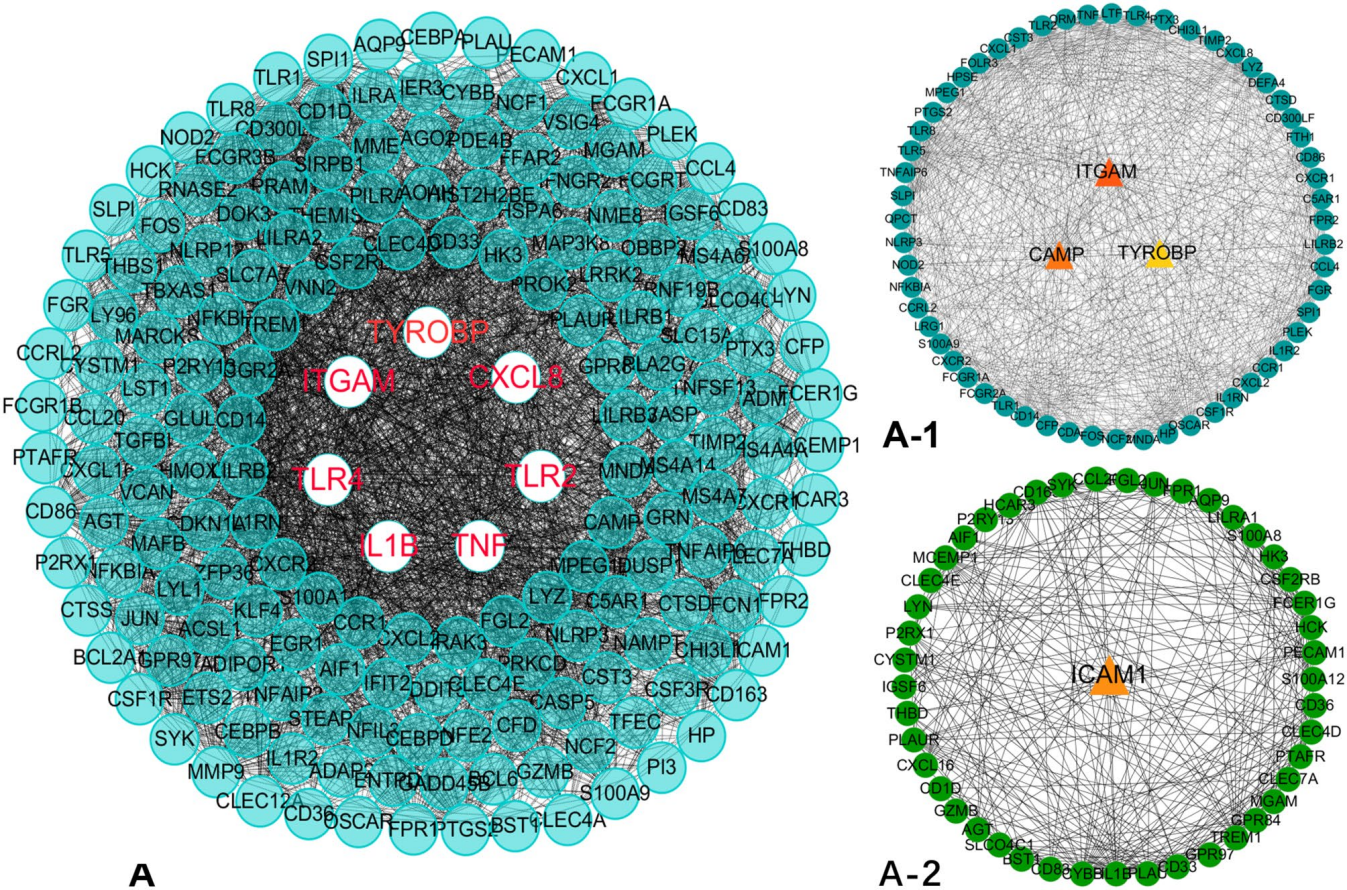
PPI network construction and identification of hub genes. (**A**) PPI network of genes in magenta module. The edge shows the interaction between two genes. Significant modules identified from the PPI network using the MCODE with a score > 6.0. (**A-1**) Molecular-1 with MCODE score = 26.2. (**A-2**) Molecular-2 with MCODE score = 13.4.

The STRING online tool was used to formulate a PPI network comprising 309 nodes and 3165 edges. Subsequent analysis found that only two MCODEs with scores > 6 were detected. Hub genes *ITGAM* (degree = 39), *CAMP* (degree = 37), and *TYROBP* (degree = 28) were identified in Molecular-1 (A-1), and *ICAM1* (degree = 18) was identified in Molecular-2 (A-2) (Fig. 6). It can be concluded that the identified genes are strongly linked to CAD.

### Results of meta-analysis based on three eligible microarrays

The meta-analysis included three different datasets from the GEO database and included a total of 80 acute myocardial infarction (AMI) and 67 normal subjects. The integrated gene expression profile was obtained after eliminating the batch effects between three datasets and also shown in Supplementary Table S6A,B. A fixed effect model was used to identify DEGs in INMEX. As a result, a total of 2409 DEGs (1125 upregulated and 1284 downregulated genes) were identified in AMI compared with normal subjects (Supplementary Table S7). Additionally, *ITGAM*, *CAMP*, *TYROBP* and *ICAM1* were all included in the upregulated DEGs group (Table 1). The expression pattern of *ITGAM*, *CAMP*, *TYROBP* and *ICAM1* across three eligible datasets are also shown in Supplementary Fig. S4. Expressions of *ITGAM*, *CAMP*, *TYROBP* and *ICAM1* genes were also markedly raised in individuals with CAD in comparison to healthy subjects.

**Table 1:** Four upregulated DEGs (*ITGAM*, *CAMP*, *TYROBP* and *ICAM1*) in CAD relative to normal subjects.

Entrez ID	Gene symbol	Gene name	Combined ES	*P* value
3684	ITGAM	Integrin subunit alpha M	1.0900	1.83E−05
820	CAMP	Cathelicidin antimicrobial peptide	1.0255	7.24E−07
7305	TYROBP	Transmembrane immune signaling adaptor TYROBP	1.1046	7.40E−08
3383	ICAM1	Intercellular adhesion molecule 1	1.0241	1.16E−06

### Validation analysis by RT-qPCR

**Figure 7:**
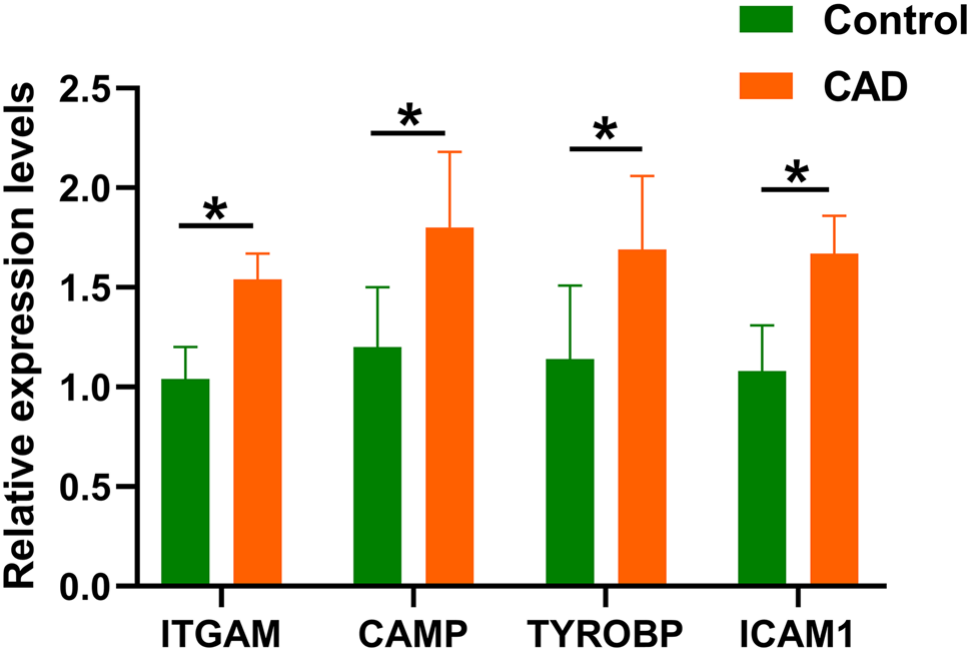
Four identified hub genes were verified by RT-qPCR. *P < 0.001.

The results of RT-qPCR uncovered that *ITGAM*, *CAMP*, *TYROBP* and *ICAM1* expressions were markedly raised in those with CAD in contrast to individuals without the condition. These findings reflect the results of the microarray analysis (Fig. 7).

### ROC curve for CAD patients

**Figure 8:**
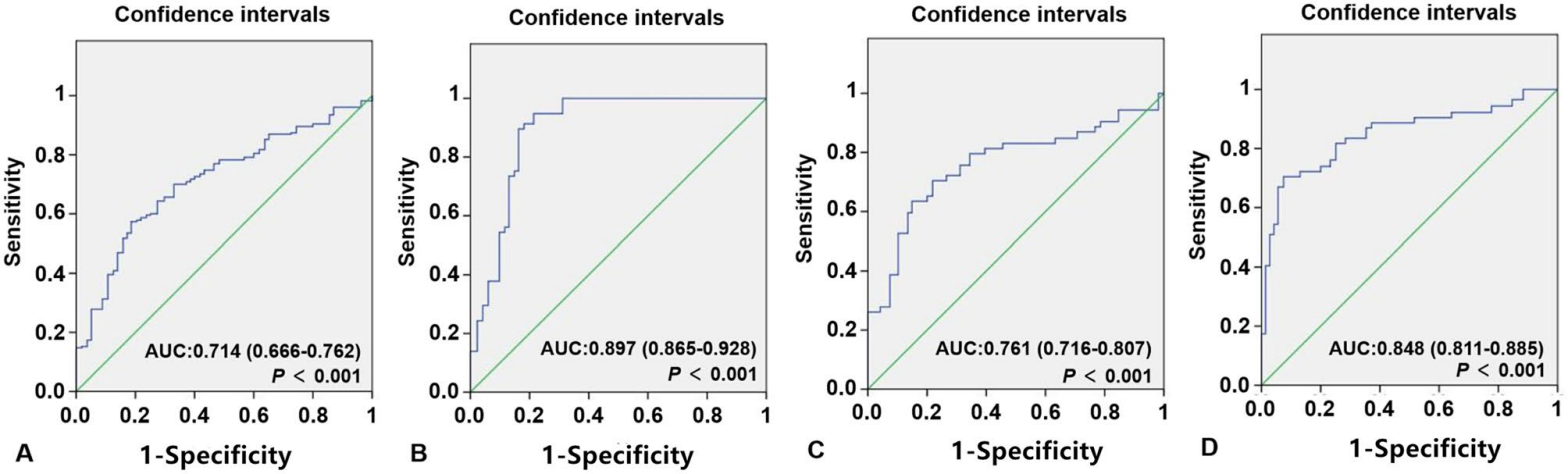
The ROC curves for the predictive values of ITGAM, CAMP, TYROBP and ICAM1 to identify CAD patients from healthy controls. (**A**) The AUC of ITGAM in CAD was 0.714 with a sensitivity of 74.3% and a specificity of 76.2%. (**B**) The AUC of CAMP in CAD was 0.897 with a sensitivity of 81.2% and a specificity of 91.1%. (**C**) The AUC of TYROBP in CAD was 0.761 with a sensitivity of 77.1% and a specificity of 79.7%. (**D**) The AUC of ICAM1 in CAD was 0.848 with a sensitivity of 83.3% and a specificity of 86.7%.

As shown in Fig. 8, ROC analysis was used to evaluate the predictive values of *ITGAM*, *CAMP*, *TYROBP* and *ICAM1* for CAD. The AUC values of *ITGAM*, *CAMP*, *TYROBP* and *ICAM1* were 0.714 (95% CI 0.666–0.762; *P* < 0.001) with a sensitivity of 74.3% and a specificity of 76.2%; 0.897 (95% CI 0.865–0.928; *P* < 0.001) with a sensitivity of 84.2% and a specificity of 91.1%; 0.761 (95% CI 0.716–0.807; *P* < 0.001) with a sensitivity of 77.1% and a specificity of 79.7% and 0.848 (95% CI 0.811–0.885; *P* < 0.001) with a sensitivity of 83.3% and a specificity of 86.7% for prediction of CAD risk, respectively. *CAMP* diagnostic performance is depicted to be superior in comparison to other genes.

### Comparison with other methods

Machine learning, more specifically, deep learning, is one of the most widely used forms of artificial intelligence. As is know that deep learning (DL) is widely used for intelligence medicine to assistant disease risk prediction and disease diagnosis based on small sample size, like transcriptomic^16^ or genomic^17^ data, and imaging data^18^. The application of deep learning in disease detection or diagnosis is particularly important for clinician, as it has the potential to maximize diagnostic performance. Previous research has demonstrated that compared with the traditional Cox model, the risk stratification model based on gene co-expression network and DL would apply deep Convolutional Neural Network (CNN) to high-dimensional gene expression data, and could improve the risk stratification and survival prediction ability of the model^19^. In addition, Liu, et al. also noticed that the multi-model deep learning framework based on CNN has better performance in the diagnosis of Alzheimer's disease (AD) and mild cognitive impairment (MCI) than the single-model method and several other competing methods^18^. However, the construction of deep learning model is tedious and time-consuming, and it is very challenging to build models with deep learning method for some parts with complex structure and difficult to image. Therefore, in the current research, the empirical ROC curves of *ITGAM*, *CAMP*, *TYROBP* and *ICAM1* were plotted using non-parametric method^20^ by SPSS (Version 22.0) software. Non-parametric method, which do not require any assumptions about data distribution, can be calculated without fitting any ROC curves^21^. Thus, the non-parametric method of area estimation under the ROC curve can be used to evaluate the accuracy of all diagnostic tests because there are no restrictions. However, the diagnostic performance of non-parametric method may be worse than that of a deep learning model.

### Demographic and biochemical characteristics

Both control and CAD individuals had similar proportions of height, age, gender ratio, and proportion of drinkers (Table 2). Patients with CAD were more likely to be smokers, and possessed higher levels of serum LDL-C, ApoB, TG and TC levels, body mass index (BMI), lucose level, weight, systolic and diastolic blood pressure as well as, glucose level, pulse pressure, in contrast to healthy individuals. Serum HDL-C and ApoA1 levels and the ApoA1/ApoB ratio were significantly higher in the control group.

**Table 2:** Comparison of demographic, lifestyle characteristics and serum lipid levels of the participants.

Characteristic	Control(n = 216)	CAD (n = 230)	Test‑statistic	*P*
Male/female	150/66	169/61	0.890	0.345
Age (years)	54.02 ± 11.66	53.17 ± 10.19	0.931	0.352
Height (cm)	164.40 ± 7.5	165.22 ± 6.97	− 1.227	0.221
Weight (kg)	58.70 ± 9.08	66.08 ± 10.73	− 7.820	3.89E−14
BMI (kg/m^2^)	20.00 ± 3.77	24.14 ± 3.23	− 12.460	9.09E−31
Smoking, *n* %	71 (32.9)	98 (42.6)	4.489	0.034
Alcohol, *n* %)	57 (26.4)	62 (27.0)	0.018	0.892
SBP (mmHg)	130.88 ± 18.75	136.74 ± 22.56	− 2.974	0.003
DBP (mmHg)	79.95 ± 11.04	82.54 ± 12.05	− 2.221	0.027
PP (mmHg)	50.93 ± 13.40	54.19 ± 16.66	− 1.974	0.049
Glu (mmol/L)	6.12 ± 1.64	6.47 ± 1.86	− 2.104	0.036
TC (mmol/L)	4.49 ± 1.02	4.74 ± 1.36	− 2.164	0.031
TG (mmol/L)	1.36 ± 1.17	1.61 ± 1.07	− 2.864	0.008
HDL-C (mmol/L)	1.65 ± 0.47	1.13 ± 0.30	13.861	1.47E−36
LDL-C (mmol/L)	2.79 ± 0.97	3.06 ± 1.11	− 2.713	0.007
ApoA1 (g/L)	1.42 ± 0.34	0.99 ± 0.31	14.057	2.18E−37
ApoB (g/L)	0.88 ± 0.21	0.95 ± 0.27	− 3.146	0.002
ApoA1/ApoB	1.69 ± 0.52	1.12 ± 0.49	11.732	7.13E−28

*SBP* Systolic blood pressure; *DBP* Diastolic blood pressure; *PP* Pulse pressure; *Glu* Glucose; *HDL-C* high-density lipoprotein cholesterol; *LDL-C* low-density lipoprotein cholesterol; *Apo* Apolipoprotein; *TC* Total cholesterol*; TG* Triglyceride.

^a^Continuous data were presented as means ± SD and determined by two side *t*-test.

^b^A Chi-square analysis was used to evaluate the difference of the rate between the groups.

## Discussion

Despite the plethora of information available regarding CAD, little is known regarding the feasibility of non-invasive diagnostic markers for this debilitating disease^22–24^. To facilitate improved treatment and diagnosis, there needs to be a deeper understanding on the underlying pathophysiology of CAD. Differential gene analysis based on microarray expression data is helpful for us to identify susceptibility genes and elucidate the molecular mechanism of CAD, however, microarray expression data are not always reproducible or are too sensitive to errors^8^. Therefore, the integration of gene expression profile data combined with WGCNA analysis may be an effective method to identify susceptibility genes of CAD. To meet this need, we integrated three different datasets from CAD patients (GSE66360, GSE19339 and GSE97320) in order to carry out WGCNA analysis, which subsequently identified 2 modules (green-yellow and magenta) that were significantly correlated with CAD. Refer to previous researches^25,26^, to determine the reliability of the identified CAD-related modules (green-yellow and magenta), we have conducted a preservation analysis of the expression profiles of CAD and we noticed that the green-yellow and magenta modules were highly preserved. Furthermore, KEGG and GO gene enrichment analyses of these two modules highlighted that those of the magenta module may impart significant biological functions closely related to inflammation, the immune response, and white blood cell activation and migration. Four hub genes (*ITGAM*, *TYROBP*, *ICAM1* and *CAMP*) were identified in two moleculars that were detected by MCODE by analyzing the PPI protein interaction network. Moreover, network-based meta-analysis revealed that the expression levels of *ITGAM*, *CAMP*, *TYROBP* and *ICAM1* in CAD patients in GSE60993 and GSE66360 datasets were significantly higher than those in the control group, at the same time, the gene expression levels of *ITGAM*, *CAMP* and *TYROBP* in CAD patients in GSE61144 dataset were also significantly higher than those in the control group. Similarly, our RT-qPCR results strongly correlated with the above results. *ITGAM*, *TYROBP*, *ICAM1* and *CAMP* gene expressions were noted to be raised in individuals with CAD in comparison to those without. Therefore, the identified *ITGAM*, *TYROBP* and *ICAM1* and *CAMP* genes were concluded to be related to CAD onset, but the underlying molecular mechanisms of these genes might be slightly different.

A recent study has proven that the occurrence of CAD is caused by a variety of factors, which is a result of interaction between alterations in plasma lipid levels, lifestyle, environmental factors and genomic background^27^. Atherosclerosis is generally regarded as the pathological foundation of CAD^6^. Atherosclerosis is a combination of abnormal lipid metabolism and a chronic inflammatory process^28^. Transendothelial migration and subintimal aggregation of monocytes are some of the most important features of early human atherosclerotic lesions. After the differentiation into macrophages and the ingestion of lipids, which causes the formation of foam cells, the arterial wall may develop atherosclerotic plaques composed of foam cells, calcium, lipids, and other components^29^. Activated leukocytes can promote vascular endothelial injury and inflammatory response, and secrete a series of inflammatory factors, such as interleukin-1 (*IL-1*), *TNF-α* and *IL-6*, resulting in cellular adhesion, infiltration of inflammatory cells, matrix degradation, all of which culminates in plaque rupture, thereby accelerating the progression of atherosclerosis^30^. Through a comprehensive search of the NCBI GENE database, we discovered that *ITGAM* (also known as *CR3A*; *MO1A*; *CD11B*; *MAC-1*; *MAC1A*; and *SLEB6*; gene ID: 3684, HGNC: 6149, OMIM: 120980) is located on chromosome 16p11.2 (exon count: 31) and encodes the integrin αM chain, which plays a crucial role in several inflammatory reactions, including the monocyte and neutrophil adhesion to damaged endothelial cells and transendothelial migration, and integrin αM is also involved in CD40L-mediated inflammation during atherosclerosis^31^. Several novel studies also proved that *ITGAM* could act as a complement component 3 receptor that is involved in the inflammatory response^32,33^. Additionally, Ayari et al. suggested that *ITGAM* and *TYROBP* expression levels were raised in human carotid artery plaques^34^. Yongming Pan et al. also found similar expression trends for *ITGAM* and *TYROBP* in a novel Tibetan minipig atherosclerosis model^35^. *TYROBP* (also known as *DAP12*; *KARAP*; *PLOSL*; *PLOSL1*; gene ID: 7305, HGNC: 12449, OMIM: 221770) encodes a transmembrane signaling polypeptide and is a type of transmembrane receptor that is ubiquitously found in macrophages/monocytes, natural killer (NK) cells and neutrophils. In recent years, NK cells, especially NKT cells, have been considered to be important participants in inflammatory cells chemotaxis, adhesion between inflammatory cells and endothelial cells, and other processes that are active in the early stages of atherosclerosis^36,37^. Previous research has demonstrated that *TYROBP* acts as one of the key drivers of a variety of inflammatory pathways^38^. Wang et al. revealed that APOE mice demonstrated plaques which richly expressed *TYROBP*, a feature thought to result in *TREM-1*/*DAP12* pathway-mediated accelerated atherosclerosis progression^39^.

A central tenet of the inflammatory process involves endothelial cell binding by leukocytes through integrins. Intercellular adhesion molecule 1 (*ICAM1*; *CD54*) is a representative ligand of integrin that is key to mediating leukocyte adhesion to the endothelial cell surface^40^. *ICAM1*-mediated endothelial chemokines attract and activate leukocytes, leading to a severe inflammatory response^41^. Silvia Dragoni et al. proved that *ICAM-1*-mediated intra-endothelial signaling plays a critical role in regulating lymphocyte transendothelial migration and modulating vascular permeability, thereby propagating chronic endothelial inflammation^42^. In addition, activation of *ICAM-1* also increased the expression of inflammatory genes correlated with coronary heart disease, such as *IL-1B*^40^, *CXCL8*, *CCL5*^41^, and *VCAM-1*^43^. Similar results were also confirmed in KEGG pathways and GO enrichment analysis, we noticed that *ITGAM*, *TYROBP* and *ICAM1* were mainly involved in the following inflammation-related signaling pathways and biological processes: leukocyte transendothelial migration (*ITGAM* and *ICAM1*), Staphylococcus aureus infection (*ITGAM* and *ICAM1*), rheumatoid arthritis (*ICAM1*), integrin-mediated signaling pathway (*ITGAM*), Toll-like receptor 4 signaling pathway (*ITGAM*), tuberculosis (*ITGAM*), natural killer cell-mediated cytotoxicity (*ICAM1* and *TYROBP*), NF-kappa B signaling pathway (*ICAM1*), and TNF signaling pathway (*ICAM1*). Therefore, we speculated that *ITGAM*, *TYROBP* and *ICAM1* may be involved in atherosclerosis by mediating the inflammatory pathways described above.

Previous studies have shown that CAD and several autoimmune diseases, such as psoriasis^44^, systemic lupus erythematosus^45^, and rheumatoid arthritis^46^, share a common pathogenesis, which suggests that the development of atherosclerosis is highly dependent on autoimmunity. The protein encoded by *CAMP* belongs to the antimicrobial peptide group that was previously established to be an autoantigen in psoriasis which is involved in cell chemotaxis, inflammatory response regulation and immune mediator induction^47,48^. Several compelling studies also proved that there was abnormal expression of *CAMP* in atherosclerotic plaques and suggested that the autoimmune response mediated by *CAMP* may be related to the development of atherosclerosis^49,50^, and these processes may partially account for the significantly raised CAD risk in patients with psoriasis^51^. Additinoally, Peter M et al*.* further confirmed that *CAMP* was a potential autoantigen implicated in the atherosclerotic immune response^52^. Furthermore, previous studies have proven that *CAMP* has been defined as a pro-atherosclerotic molecule^53,54^ and that *CAMP-*deficient transgenic mice have a reduced risk of atherosclerosis^55^. In addition, we noticed that *CAMP* was mainly enriched in the following signaling pathways and biological processes: tuberculosis, innate immune response, cell redox homeostasis, cellular response to interleukin-1, and cellular response to tumor necrosis factor. These findings indicated that *CAMP* may be involved in atherosclerosis by mediating autoimmune or inflammatory responses. Furthermore, the independent verification data as well as our RT-qPCR results also revealed that CAD patients had significantly raised *ITGAM*, *TYROBP*, *ICAM1* and *CAMP* expression levels in contrast to healthy subjects. In addition, based on ROC curve analysis, we propose *CAMP* to function as a potential diagnostic biomarker for CAD.

This research had several limitations. Firstly, we have only identified and verified the hub genes that were associated with CAD, but have not constructed the transcriptional regulatory network, which may lead to more meaningful discoveries. Secondly, the sample sources of the three datasets selected in the current research are different and the biological differences will inevitably produce an impact on our findings. Thirdly, there was only one disease phenotype of clinical features in this study, thus, more clinical features are needed in order to further define the phenotype-genotype relationship. Fourthly, this is a single-center study comprising of a small patient number, and large multi-center studies are necessary validate our findings. Lastly, the molecular mechanisms of *ITGAM*, *TYROBP*, *ICAM1* and *CAMP* involved in CAD are still not fully defined and require further cytology and animal experiments to further outline their respective roles in vivo and in vitro.

In summary, we determined that *ITGAM*, *TYROBP*, *ICAM1* and *CAMP* may possess significant roles in mediating the chronic inflammatory process that eventually culminates in atherosclerosis and CAD. The underlying mechanism may be related to transendothelial migration of leukocytes and the immune response. Independent verification data, combined with our RT-qPCR results were similar to those derived from the microarray analysis, which further increased the credibility of the conclusion.

## Materials and methods

### CAD microarray data sets were used to identify hub genes

Three microarray data sets originating from individuals with CAD (GSE66360, GSE19339 and GSE97320) were extracted from the National Center for Biotechnology Information (NCBI) Gene Expression Omnibus (GEO, http://www.ncbi.nlm.nih.gov/geo/) database. This data was based on the GPL570 Affymetrix Human Genome U133 Plus 2.0 array and was used to construct a co-expression network. The expression profile data from 56 CAD samples and 57 normal samples across three data sets were analyzed using integrated analysis. Before we analyzed the data, some powerful and accurate R packages, such as affy, affyPLM, and RColorBrewer were used to perform quality testing of microarray data. Some functions in affyPLM package can be used to fit the original data of microarray and generate the weights and residuals diagram, relative the relative log expression (RLE) and the relative standard deviation (NUSE, Normalized unscaled standard errors) box diagram^25^. After confirming that there are no outliers, RMA methods were used to normalize gene expression value matrices which were extracted from the original files in CEL format. Furthermore, KNN function of the impute package is used to calculate and supplement missing values. SVA methods were then used to remove and batch differences via the R software (version 4.0.0)^12^. After this, gene symbols were designated based on probe identification numbers (IDs) using the Bioconductor package^13^. Average expression values were used in cases where multiple probe IDs corresponded to the same gene.

### Construction of the weighted gene co-expression network

WGCNA is a widely used systems biology method that is able to transform gene expression data profiles into a scale-free network^9^. Outlier samples were excluded to maintain the reliability of network construction results. The appropriate soft threshold power (soft power = 14) was chosen with reference standard scale-free networks, with the power function used to calculate adjacency values between all differentially expressed genes. A topological overlap matrix (TOM) was then formulated based on the adjacency values in order to calculate the corresponding dissimilarity (1-TOM) values. Module identification was accomplished with the dynamic tree cut method by hierarchically clustering genes using 1-TOM as the distance measure with a minimum size cutoff of 30 and a deep split value of 2 for the resulting dendrogram. A module preservation function was used to verify the stability of the identified modules by calculating module preservation and quality statistics in the WGCNA package^14^.

### Preservation analysis of five network modules

Refer to the methods described in a previous study^26^, a composite preservation statistics method based on the module Preservation function in the WGCNA R package was used to verify the conservativeness of the five modules. The Z-summary statistic was used to measure module density and intramodular connectivity metrics in each module. In the corresponding network, Zdensity (function 1) was used to conducted the 4 density preservation statistics, Zconnectivity (function 2) was used to conducted the 3 connectivity-based statistics, the combines module density and intramodular connectivity metrics was measured by the Zsummary (function 3) and defined as follows: Z density = median (Z meanCor, Z meanAdj, Z propVarExpl, Z meanKME) (function 1); Z connectivity = median (Z cor.kIM, Z cor.kME, Z cor.cor) (function 2); Z summary = (Z density + Z connectivity)/2 (function 3). In addition, if Z summary < 2 indicated no evidence that the module preserved; if 2 < Z summary < 10 indicated weak to moderate preservation; if Z summary > 10 indicated high preservation among modules. The module size has a strong influence on Z statistics. Therefore, the medianRank for preservation analysis was conducted to comparing the preservation statistics of different sized modules. It indicates that modules with lower median rank tend to show better preservation statistics than those with higher median rank.

### Identification of the module of interest and functional annotation

The relationship between clinicopathological characteristics and modules were discerned using the Pearson correlation analysis in order to determine CAD-related biological modules. Significant gene modules were subsequently processed with the Gene Ontology (GO) and Kyoto Encyclopedia of Genes and Genomes (KEGG) pathway analyses by using the Database for Annotation, Visualization and Integrated Discovery (DAVID) online tool (version 6.8; http://david.abcc.ncifcrf.gov). *P* < 0.05 was set as the cutoff criterion.

### Hub gene analysis

The degree of module membership (MM) was defined as the correlation between module eigengenes (Mes) and gene expression profiles. The correlation between the gene and external traits was determined to be the degree of gene significance (GS). Generally, identified modules with increased MS and GS values were then subjected to further scrutinization for their biological function^15^. The Search Tool for the Retrieval of Interacting Genes database (version 11.0; http://www.string-db.org) was used to construct protein–protein interaction (PPI) gene networks based on the chosen module^16^. This network was then visualized with the Cytoscape software^17^, with the most valuable clustering module identified using molecular complex detection (MCODE)^18^. Modules with an MCODE score > 6 were selected for further analysis.

### Sample verification and diagnostic criteria

Two hundred thirty unrelated patients with CAD were recruited from the Shao Yang Central Hospital. CAD was defined as significant coronary artery stenosis (≥ 50%) in at least one of the three main coronary vessels or their main branches (branch diameter ≥ 2 mm)^19^. The diagnostic criteria for the three types of CAD patients are as follows: (i) stable exertional angina (n = 122), defined as episodes with reversible ischemic chest pain. (ii) Non-ST-elevation acute coronary syndromes (NSTE-ACS) that included non-ST‐elevated myocardial infarction (NSTEMI) patients and unstable angina (n = 66), defined as angina at crescendo or rest angina. (iii) ST‐elevated myocardial infarction (STEMI) (n = 42) defined as elevated plasma levels of Troponin T (TnT; at least one value above the 99th percentile) together with ST-segment elevation or new left bundle branch block in the electrocardiogram and ischemic symptoms. All subjects had no history of autoimmune, thyroid, renal, neoplastic, hematologic, liver disease or type 1 diabetes. The control group consisted of two hundred sixteen healthy controls matched by age, ethnicity (Han Chinese) and gender and were randomly recruited from the Physical Examination Center of the Shao Yang Central Hospital, in the same period. All control individuals were assessed with questionnaires, clinical history and examination to ensure the absence of type 2 diabetes mellitus (T2DM), previous myocardial infarction or CAD as well as ischemic stroke (IS). Written, informed consent was gained from all individuals prior to participation and all experiments were performed in accordance with relevant named guidelines and regulations. The research proposal was approved by the Ethics Committee of the Shao Yang Central Hospital (No: KY 2020-023-08).

### Network-based meta-analysis to verify the identified hub genes

Acute myocardial infarction (AMI) samples from three datasets (GSE60993, GSE61144 and GSE66360) were selected to verify the identified hub genes, which are based on the platform of GPL6884 Illumina HumanWG-6 v3.0 expression beadchip, GPL6106 Sentrix Human-6 v2 Expression BeadChip and GPL570 Affymetrix Human Genome U133 Plus 2.0 array and used to construct the co-expression network. Raw data (.CEL) were processed using software package in R (version 4.0.0) for microarray quality assessment such as RNA degradation plots, normalized unscaled standard errors (NUSE) and relative log expression (RLE). Several samples with abnormal distribution in each dataset were removed. A robust multi-array average (RMA) algorithm with background adjustment, log transformation and normalization was used to pre-process all data^56^. The Bioconductor package was used to transform the probe identification numbers (IDs) into gene symbols^13^. Average expression values were used in cases where multiple probe IDs corresponded to the same gene. INMEX was used to carry out microarray-based meta-analysis in order to incorporate multiple gene expression datasets^57^, in compliance to the Preferred Reporting Items for Systematic Reviews and Meta-Analyses guidelines for meta-analysis^58^. Each eligible gene expression profile was uploaded to INMEX. After each gene expression profile integrity check, the ComBat option in INMEX was used to eliminate batch effects between three different gene expression profiles using empirical byes methods^59^. To eliminate the differences in platform usage and study design, heterogeneity among microarray datasets, a fixed effect model (FEM) was selected for the meta-analysis in compliance to the between-study heterogeneity based on Cochran's Q test^60^. Differentially expressed genes (DEGs) from the integrated dataset were obtained by the GeneVenn web tool from INMEX^61^.

### Quantitative real-time PCR

Total RNA was extracted from isolated peripheral blood monocytes (PBMCs) using the TRIzol reagent. cDNA was then reverse-transcribed with the PrimeScript RT reagent kit (Takara Bio, Japan). RT-qPCR was then carried out with the resultant cDNA as a template. Hub gene-specific primers used in these experiments were designed by Sangon Biotech (Shanghai, China) and are detailed in Table 3. An ABI Prism 7500 sequence-detection system (Applied Biosystems, USA) using a Taq PCR Master Mix Kit (Takara) was used to perform quantitative RT-PCR was performed using RT Reaction Mix in a total volume of 20 μL with the following reaction conditions: predenaturation at 95 °C for 30 s, then 40 cycles of 95 °C for 30 s and 60 °C for 30 s.

**Table 3:** PCR primers for quantitative real-time PCR.

Gene	Forward primer	Reverse primer
ITGAM	TCCCGGAAAACTCAGAGGTC	TGAGGCCGTGAAGTTGAGAT
CAMP	AGGTACTGTGGAAAGCCTGC	GACCCATTGGATGGTCCACA
TYROBP	CCTCAACTCACCACTCTGCC	TTCAAGGTTTGGGGGTGCTT
ICAM1	TCCTCACCGCCTGTTGTATC	ACTTCCCCTCTCATCAGGCT
GAPDH	AGAGAGAGGCCCTCAGTTGCT	TTGTGAGGGAGATGCTCAGTGT

### Diagnostic criteria

Serum triglyceride (TG; 0.56–1.70 mmol/L), ApoA1 (1.20–1.60 g/L), total cholesterol (TC; 3.10–5.17 mmol/L), apolipoprotein (Apo) B (0.80–1.05 g/L), low-density lipoprotein cholesterol (LDL-C; 2.70–3.10 mmol/L) and high-density lipoprotein cholesterol (HDL-C; 1.16–1.42 mmol/L) and the ApoA1/ApoB ratio (1.00–2.50) levels were defined as their respective normal values at our Clinical Science Experiment Center. The diagnostic criteria of diabetes^20^, hypertension^21^, obesity, normal weight, overweight^22^ and hyperlipidemia^23^ were based on previous studies.

### Statistical analyses

All data was analysed with the SPSS (Version 22.0). Continuous data is depicted in terms of mean ± SD. Independent-samples t tests were used to assess the general characteristics differences between controls and individuals with CAD patients and controls. The Chi-square test was utilized to evaluate the differences in the amount of alcohol consumers, age distribution and proportion of smokers between controls and individuals with CAD. Referring to previous studies^62,63^, receiver operating characteristic (ROC) curves were built based on plasma levels of *ITGAM*, *CAMP*, *YROBP* and *ICAM1* to evaluate the specificity, sensitivity, and respective areas under the curves (AUCs) with 95% CI. The optimal cut-off value for diagnosis was investigated by maximising the sum of sensitivity and specificity and minimising the overall error (square root of the sum [1 − sensitivity]^2^ + [1 − specificity]^2^), and by minimising the distance of the cutoff value to the top-left corner of the ROC curve, and the corresponding empirical ROC curve of *ITGAM*, *CAMP*, *TYROBP* and *ICAM1* were drawn by a nonparametric method using SPSS software (Version 22.0). R software (version 4.0.0) was used to carry out bioinformatic analysis and heat mapping of the correlation models.

## Supplementary Information

Supplementary Figures.

Supplementary Table S1A.

Supplementary Table S1B.

Supplementary Table S2.

Supplementary Table S3.

Supplementary Table S4.

Supplementary Table S5.

Supplementary Table S6A.

Supplementary Table S6B.

Supplementary Table S7.

Supplementary Legends.
