# Supplementary material for: Weighted gene co-expression network analysis identifies specific modules and hub genes related to coronary artery disease
Source: Sci Rep. 2021 Mar 23;11:6711. doi: 10.1038/s41598-021-86207-0 (PMC7988178; doi:10.1038/s41598-021-86207-0)
Supplement: Supplementary file 1 — Supplementary Figures. [file 41598_2021_86207_MOESM1_ESM.docx]

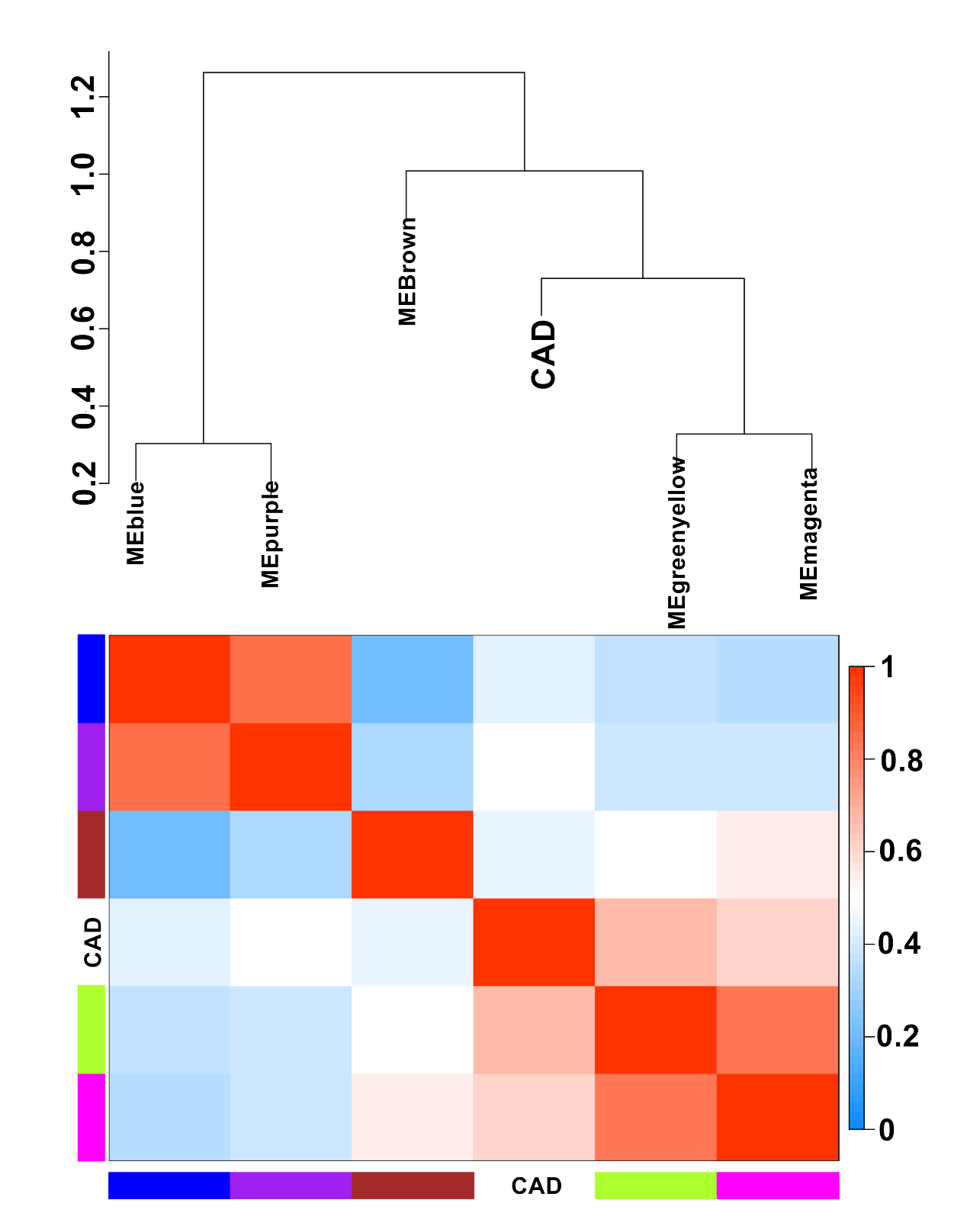


**Additional Figure S1**. **Hierarchical clustering and heatmap of module eigengenes (labeled by their colors) and the CAD**. On top was hierarchical clustering of module eigengenes that summarize the modules found in the clustering analysis. Branches of the dendrogram (the meta-modules) group together eigengenes that are positively correlated. Below was heatmap plot of the adjacencies in the eigengene network. Each row and column in the heatmap corresponds to one module eigengene (labeled by color). In the heatmap, red represents high adjacency, while blue color represents low adjacency. Squares of red color along the diagonal are the meta-modules


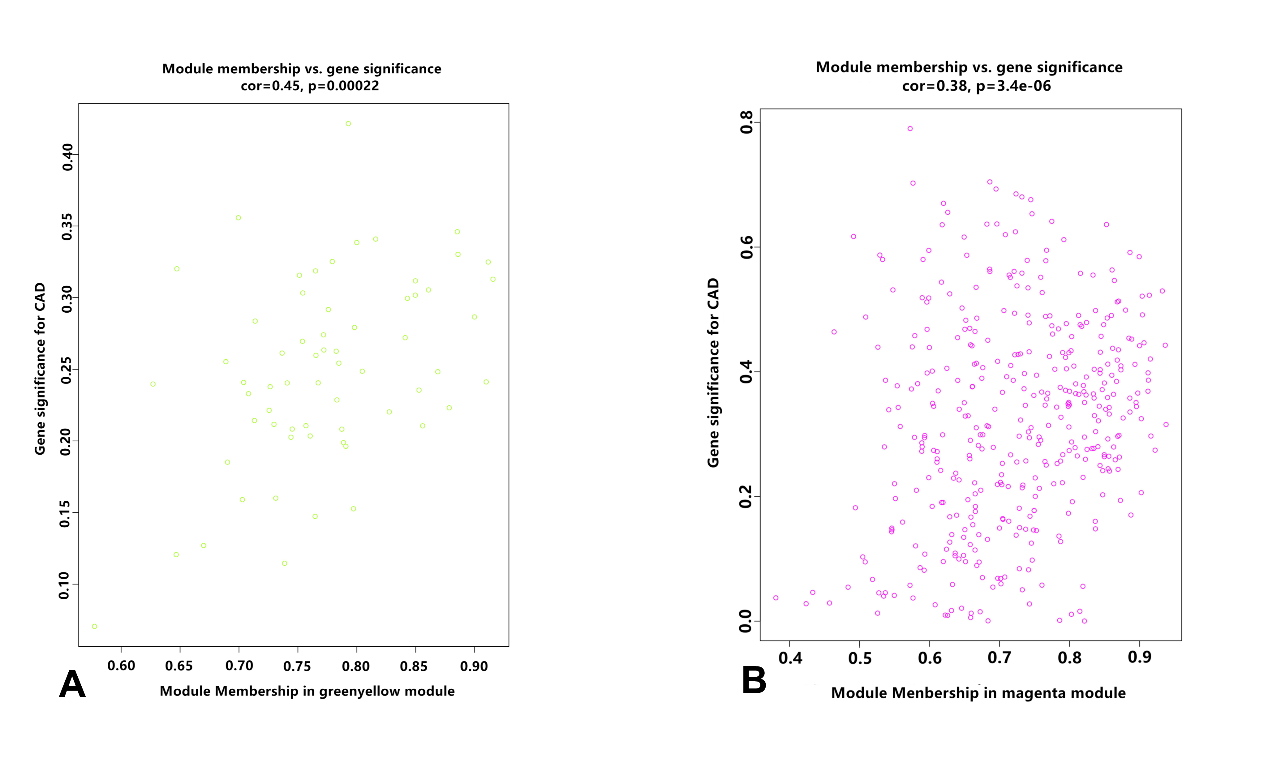


**Additional Figure S2. Module membership – gene significance correlation analyses.** Scatterplot shows a highly significant correlation between gene significant (GS) versus module membership (MM) with coronary artery disease (CAD) in the greenyellow (**A**) and magenta (**B**) modules.


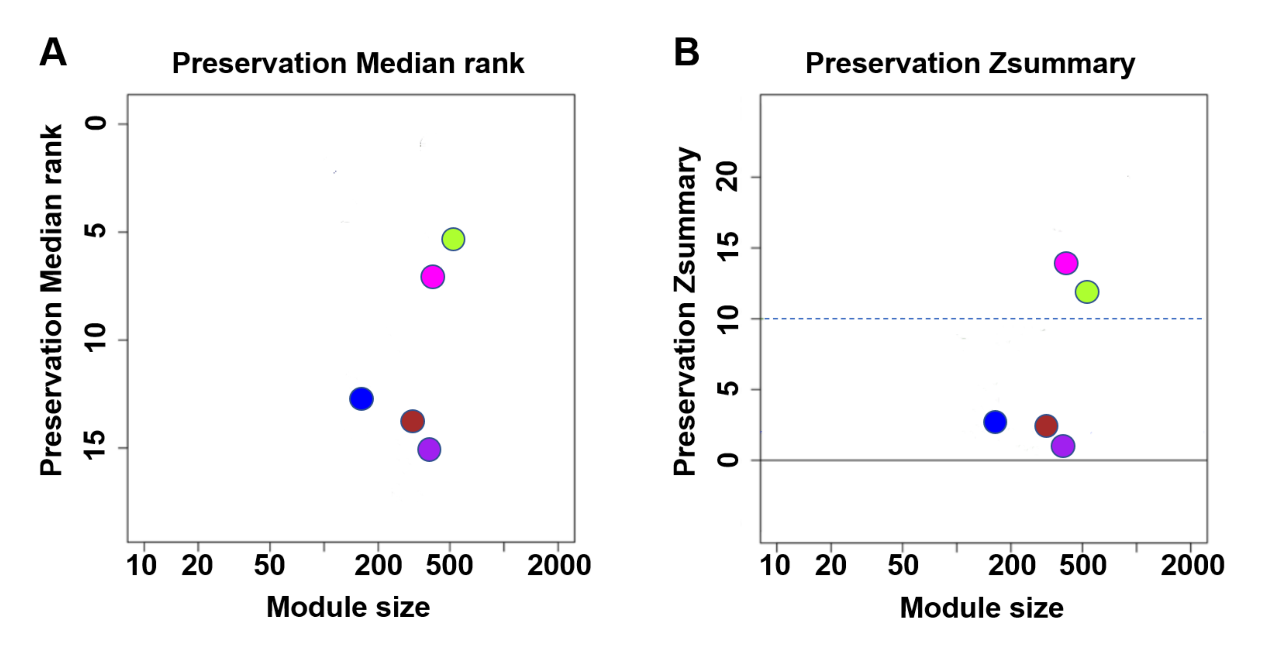


**Additional Figure S3. Preservation analysis of five network modules.** The Y-axis represents preserved values and the X-axis represents module size. (**A**) median Rank test; and (**B**) Z summary statistics test.


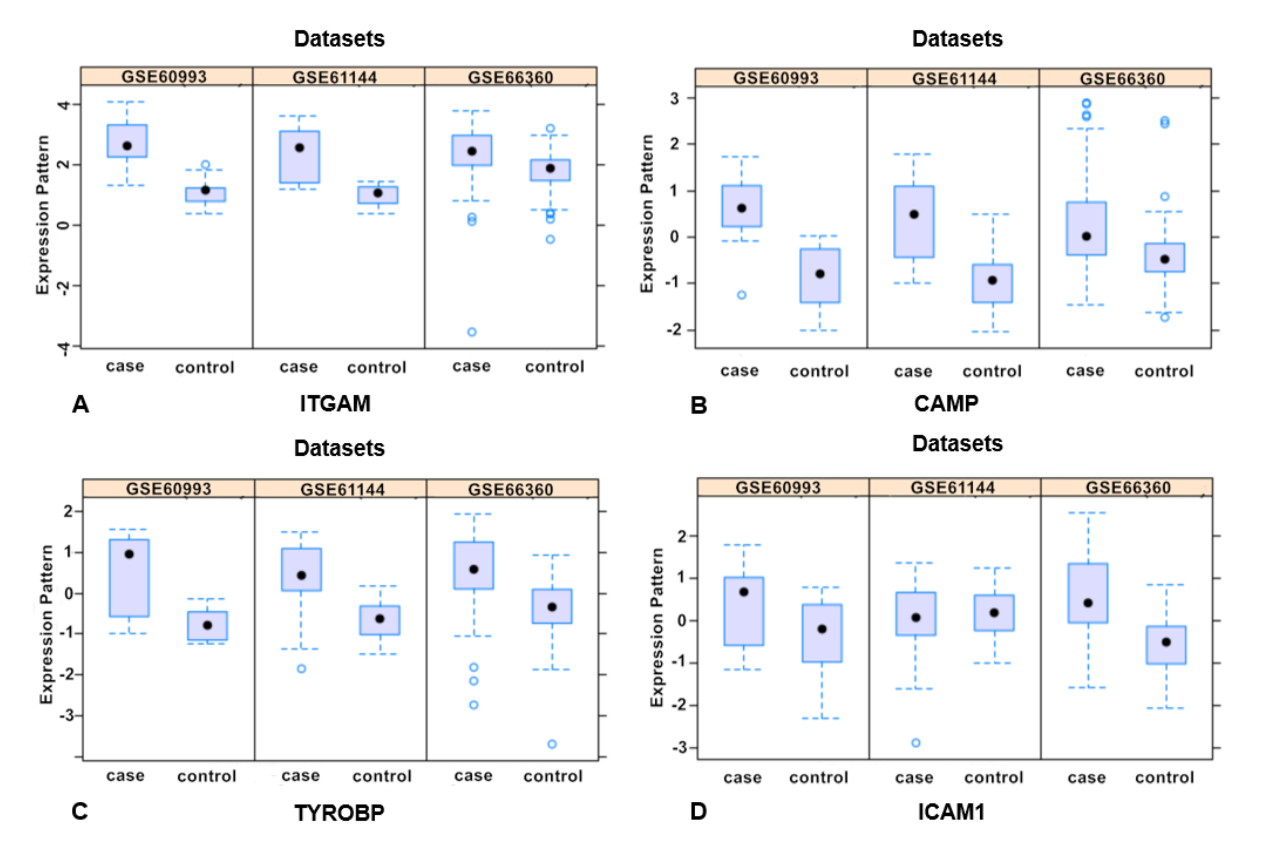


**Additional Figure S4. The expression pattern of *ITGAM*, *CAMP*, *TYROBP* and *ICAM1* in three eligible datasets.**
