# Supplementary material for: Weighted gene co-expression network analysis identifies specific modules and hub genes related to coronary artery disease
Source: Sci Rep. 2021 Mar 23;11:6711. doi: 10.1038/s41598-021-86207-0 (PMC7988178; doi:10.1038/s41598-021-86207-0)
Supplement: Supplementary file 11 — Supplementary Legends. [file 41598_2021_86207_MOESM11_ESM.docx]

**Weighted gene coexpression network analysis identifies specific modules and hub genes related to coronary artery disease**

Peng-Fei Zheng^1,2^, Lu-Zhu Chen^1^, Yao-Zong Guan^2^, Peng Liu^1^ ID:*

*Correspondence: ying_lpxm@163.com

ID: 0000-0002-3095-0352

^1^ Department of Cardiology, ShaoYang Central Hospital, 36 QianYuan lane, Shaoyang 422000, Hunan, People’s Republic of China

^2^ Graduate School of Guangxi Medical University, 22 Shuangyong Road, Nanning 530021, Guangxi, People’s Republic of China

**Additional file: Tables S1.** Gene expression profiles of 114 samples.

**Additional file: Tables S2.** Disease phenotypes of 114 samples.

**Additional file: Tables S3.** Total of 309 genes in the magenta module.

**Additional file: Tables S4.** GO functional enrichment analysis of the genes in the magenta module.

**Additional file: Tables S5.** KEGG functional enrichment analysis of the genes in the magenta module.

**Additional file: Tables S6.** The integrated gene expression profile was obtained after eliminating the batch effects between three datasets.

**Additional file: Tables S7.** A total of 2409 DEGs (1125 upregulated and 1284 downregulated genes) were identified in CAD compared with normal subjects.

**Additional Figure S1**. Hierarchical clustering and heatmap of module eigengenes (labeled by their colors) and the CAD.

**Additional Figure S2.** Module membership – gene significance correlation analyses.

**Additional Figure S3.** The expression pattern of *ITGAM*, *CAMP*, *TYROBP* and *ICAM1* in three eligible datasets.
